# The Mucinous Embolus: An Atypical Aortic Saddle Occlusion Unveiling a Silent Cardiac Threat

**DOI:** 10.7759/cureus.117981

**Published:** 2026-10-15

**Authors:** Kevin Zambrano Zambrano, Emmanuel Ortega Martinez, Aimee Castillo Zambrano, Rebeca Noemi Delgado Soto, Julio Martinez Salazar

**Affiliations:** 1 Internal Medicine, National Medical Center November 20, México City, MEX; 2 Faculty of Medicine, Universidad Autónoma de Guadalajara, Guadalajara, MEX; 3 Faculty of Medicine, Universidad Westhill, México City, MEX

**Keywords:** acute limb ischemia, aortic saddle embolus, atrial myxoma, cardiac tumor, renal infarction, showering emboli, transesophageal echocardiography

## Abstract

Atrial myxomas are rare primary cardiac tumors that typically present with obstructive symptoms but can occasionally lead to devastating embolic consequences. Massive tumor fragmentation causing acute aortoiliac occlusion, known as a saddle embolus, combined with synchronous visceral infarctions, is an exceptionally rare and life-threatening presentation. This severe multisystemic manifestation can complicate both immediate surgical management and the subsequent diagnostic focus. Bedside clinical scrutiny, specifically the macroscopic appraisal of an atypical, friable, and mucinous embolus extracted during emergency limb salvage, serves as a fundamental diagnostic compass to uncover silent cardiac sources. This highlights the critical importance of pursuing systemic multimodality imaging to detect the phenomenon of "showering emboli" even after successful peripheral reperfusion. The case discussed herein illustrates the diagnostic approach and clinical decision-making in a patient presenting with a catastrophic mucinous saddle embolus that unmasked an occult left atrial myxoma.

## Introduction

Primary cardiac tumors are exceedingly rare, with a reported incidence between 0.0017% and 0.19% in an autopsy series, of which atrial myxomas account for the vast majority [1]. Despite being histologically benign neoplasms, they often exhibit aggressive initial clinical behaviour due to their obstructive potential; depending on their size and location, symptoms range from exertional dyspnoea to acute heart failure [2]. Systemic embolic events occur in 20-40% of cases; however, limb involvement is unusual, reported in merely 2.15% of patients. The stratification of this risk is closely tied to tumour morphology: "atypical" phenotype masses, characterized by irregular contours and a soft or friable consistency, pose a significantly higher probability of fragmentation and embolization (OR: 1.45) [3]. Specifically, massive embolization resulting in acute, bilateral occlusion of the aortic bifurcation (saddle embolism) is considered an exceptionally rare event that constitutes a true surgical catastrophe [4].

## Case presentation

A 56-year-old woman with a history of hypertension and type 2 diabetes presented to the emergency department with acute critical ischemia (Rutherford category IIb) [5] in both lower extremities. Physical examination confirmed the bilateral absence of femoral and distal pulses. Initial computed tomography angiography (CTA) revealed a massive saddle embolus occluding the aortic bifurcation and a synchronous corticomedullary filling defect in the upper pole of the right kidney (Figure 1A). 

**Figure 1:**
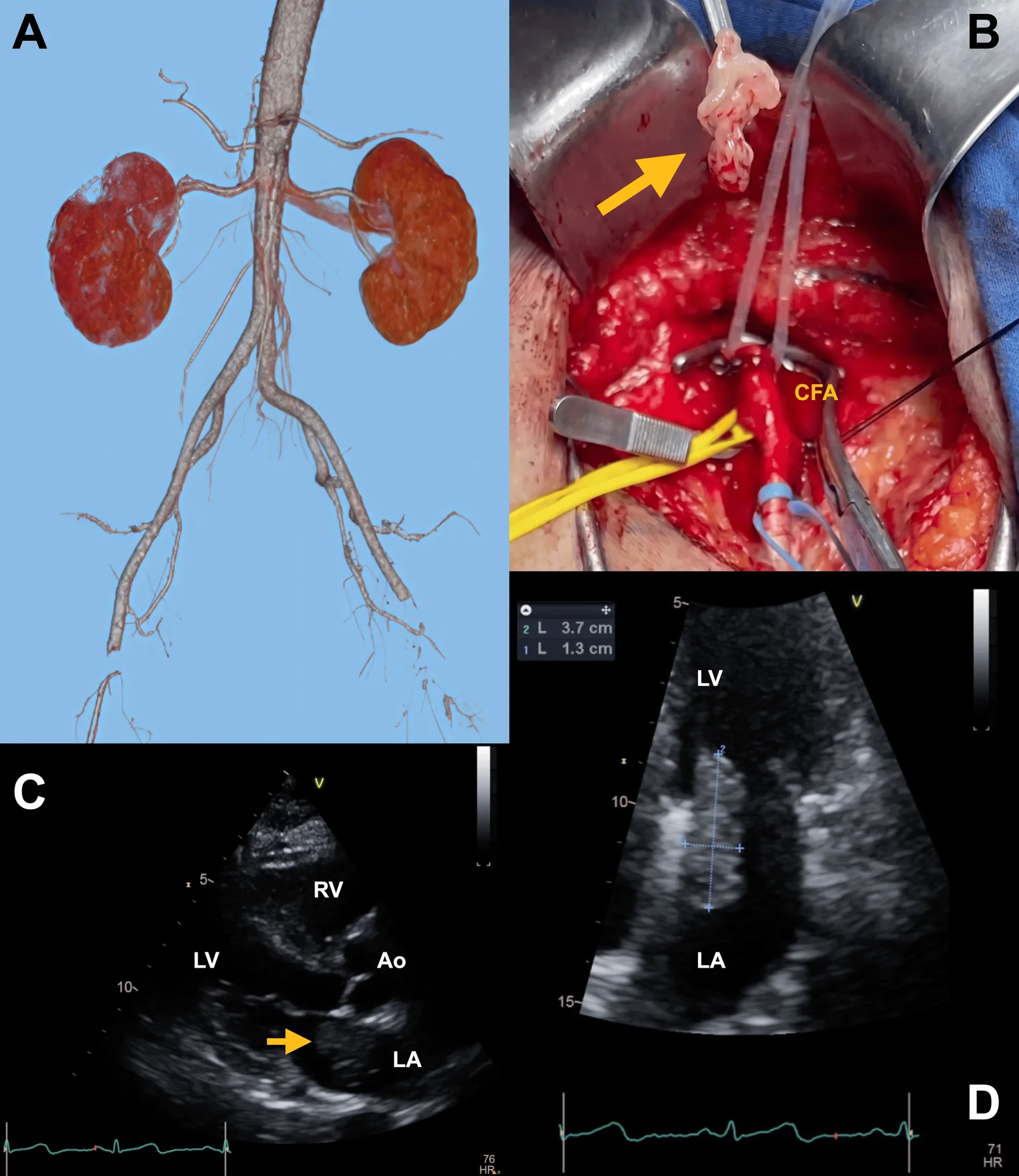
Clinical presentation and preoperative imaging (A) Three-dimensional computed tomography angiography (CTA) reconstruction demonstrating a massive aortic saddle embolus and a synchronous wedge-shaped right renal infarct. (B) Macroscopic view of the right femoral embolectomy yielding a friable, translucent mucoid fragment (yellow arrow). (C) Preoperative parasternal long-axis transthoracic echocardiogram (TTE) showing the left atrial mass prolapsing through the mitral orifice in diastole (yellow arrow). (D) Preoperative apical four-chamber TTE detailing the dimensions of the large, lobulated mass (3.7×1.3 cm) attached to the interatrial septum. CTA: computed tomography angiography; TTE: transthoracic echocardiogram

The diagnostic turning point occurred during the emergency limb-salvage intervention. An open embolectomy was performed via a right femoral approach using a Fogarty catheter. Given the extraordinary elastic cohesiveness of the obstructive material, the entire aortic embolus was successfully retrieved from this single access site; flow restoration was confirmed by vigorous retrograde bleeding and the immediate return of contralateral pulses, rendering a left groin incision unnecessary. Clearance of the aortic bifurcation and successful bilateral reperfusion were immediately confirmed intraoperatively by vigorous pulsatile retrograde bleeding, the return of palpable distal pulses, and bedside continuous-wave Doppler ultrasound. Postoperatively, the patient was initiated on a continuous unfractionated heparin infusion as a bridge to definitive cardiac surgery. However, the extracted material was friable and mucinous, morphologically distinct from a conventional red cruoric thrombus (Figure 1B). This atypical macroscopic appearance immediately alerted the team to a potential tumoural origin. Urgent histopathological analysis revealed organized thrombotic material alongside bland lepidic cells embedded within a diffuse myxoid stroma, confirming the suspicion (Figures 2A, 2B). Guided by this histological hallmark, an urgent transthoracic echocardiogram (TTE) detected a mobile mass in the left atrium prolapsing through the mitral annulus in diastole (Figure 1C).

**Figure 2:**
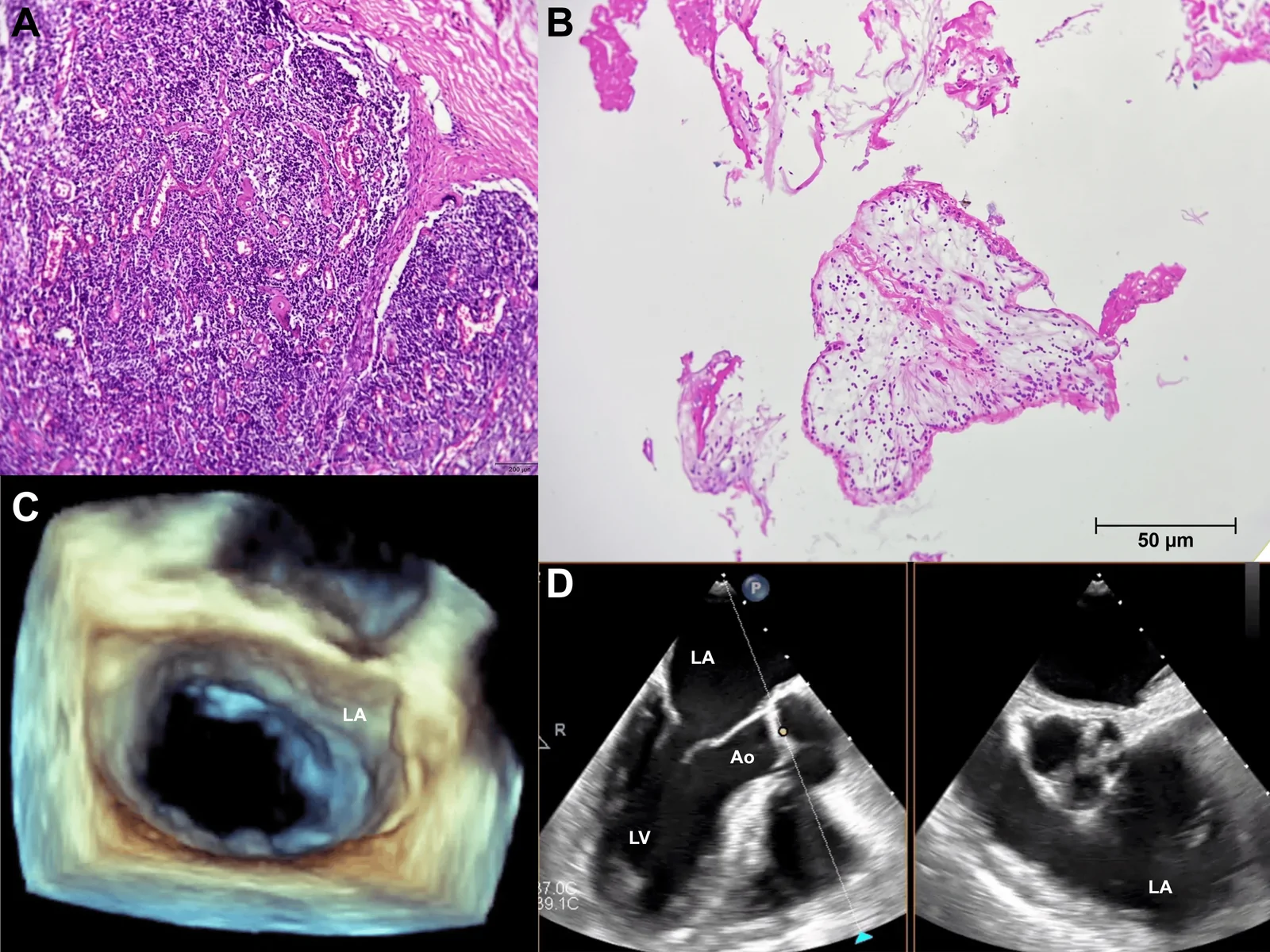
Histopathological correlation and postoperative structural resolution (A) Light microscopy (hematoxylin and eosin (H&E), x10) of the embolectomy specimen showing organized thrombotic material with dense fibrin networks and inflammatory infiltrates. (B) High-power microscopy (H&E, x40) revealing bland stellate-to-oval lepidic cells embedded within a diffuse myxoid stroma, confirming a cardiac myxoma. (C) Postoperative three-dimensional transoesophageal echocardiography (TEE) confirming a clear left atrial cavity following complete tumour excision. (D) Postoperative mid-oesophageal biplane TEE (XPlane) visualizing the intact reconstructed interatrial septum and absence of any residual mass. H&E: haematoxylin and eosin; TEE: transoesophageal echocardiography

Upon transfer to our tertiary referral centre, the diagnostic evaluation was expanded through multimodality imaging. The TTE accurately delineated a 3.7×1.3 cm lobulated tumor attached to the interatrial septum (Figure 1D). Preoperative echocardiography demonstrated preserved left ventricular ejection fraction (LVEF >60%) and moderate diastolic mitral inflow obstruction without structural valve damage. To rule out further systemic embolization, a comprehensive computed tomography of the brain and abdomen was performed, which revealed no additional ischemic territories beyond the known renal infarct.

To mitigate the compounded risk of systemic inflammatory response syndrome and severe ischemia-reperfusion injury following critical limb ischemia, definitive cardiac surgery was purposefully deferred. This ten-day interval allowed for optimal hemodynamic stabilization and continuous monitoring. Subsequently, the patient underwent successful tumour resection via median sternotomy with cardiopulmonary bypass. Complete cavity clearance and interatrial septal reconstruction were achieved, fully documented by postoperative transesophageal echocardiography (TEE) (Figures 2C, 2D), which also confirmed an intact mitral valve apparatus and preserved ventricular function. Histopathological examination of the resected atrial mass independently confirmed the diagnosis of a cardiac myxoma, correlating perfectly with the embolic specimen. The postoperative course was remarkably uneventful. Renal function remained stable and strictly within normal reference ranges throughout hospitalization, without the need for renal replacement therapy. She was discharged on postoperative day 7 and remains asymptomatic, with full lower-extremity perfusion and no echocardiographic evidence of tumor recurrence at the six-month follow-up.

## Discussion

Given their variable clinical presentation, cardiac myxomas are considered the "great masqueraders" of cardiology. Although they typically present with obstructive symptoms, limb embolism alone is infrequent, and the simultaneous occurrence in both lower extremities is exceptionally rare [4,6,7]. While embolization to visceral sites such as the renal, splenic, and mesenteric arteries is well-documented, it represents a minority of cases, with splenic infarction being the most prevalent (1.93% of embolic events). Renal artery embolization has a low incidence; therefore, the concurrent presentation of critical ischaemia in both lower limbs and visceral involvement constitutes an extraordinarily rare phenomenon [3,7].

This case underscores a vital clinical lesson: the macroscopic characteristics of an embolus can serve as the primary diagnostic guideline. In our patient, the extraction of "mucinous" material during the embolectomy was the critical trigger for immediate echocardiographic screening. Although current recommendations dictate prioritizing limb viability, a comprehensive aetiological search using multimodal imaging tools must also be advocated [8]. The finding of the right renal infarct on the initial CTA-which was clinically silent upon admission-highlights the dynamic behaviour of these tumours. While our single cross-sectional imaging cannot definitively establish the precise chronology of the events, this concurrent visceral involvement strongly raises the suspicion of an asynchronous embolic phenomenon or the so-called "showering emboli" prior to cardiac surgery, reaffirming the myxoma as a critical aetiology of acute renal infarction [9]. Furthermore, literature maintains that stromal friability and a villous or gelatinous morphology are primary determinants of embolic potential, independent of tumour size, which is usually more associated with obstructive symptoms [10].

Regarding treatment, surgical intervention remains the cornerstone and the sole curative management. The prognosis following successful resection is excellent, with survival rates exceeding 80% even at 20 years of follow-up. The recurrence rate for sporadic myxomas is notably low, reported between 1% and 3%, underscoring the efficacy of a timely surgical approach [1,11].

## Conclusions

The diagnosis of an occult cardiac myxoma presenting as a catastrophic vascular emergency relies entirely on maintaining a high index of clinical suspicion beyond standard ischemic algorithms. This case highlights a fundamental educational paradigm: the bedside macroscopic appraisal of an extracted embolus, specifically its atypical, friable, and mucinous nature, serves as a critical diagnostic clue that should prompt immediate further investigation. Furthermore, the presence of an aortic saddle occlusion combined with a synchronous silent renal infarct raises strong clinical suspicion of an asynchronous embolic mechanism, often referred to as "showering emboli". Therefore, successful peripheral reperfusion must not halt the diagnostic workup; rather, it mandates immediate systemic screening via multimodality imaging. Integrating prompt surgical embolectomy with swift echocardiographic and histopathological correlation remains the absolute cornerstone for unmasking these hidden tumors, preventing further systemic devastation, and securing curative long-term outcomes.

## References

[1] Reynen K (1995). Cardiac myxomas. N Engl J Med.

[2] He DK, Zhang YF, Liang Y, Ye SX, Wang C, Kang B, Wang ZN (2015). Risk factors for embolism in cardiac myxoma: a retrospective analysis. Med Sci Monit.

[3] Yin L, Wang J, Li W, Ling X, Xue Q, Zhang Y, Wang Z (2016). Usefulness of CHA(2)DS(2)-VASc scoring systems for predicting risk of perioperative embolism in patients of cardiac myxomas underwent surgical treatment. Sci Rep.

[4] Pinede L, Duhaut P, Loire R (2001). Clinical presentation of left atrial cardiac myxoma. A series of 112 consecutive cases. Medicine (Baltimore).

[5] Rutherford RB, Baker JD, Ernst C, Johnston KW, Porter JM, Ahn S, Jones DN (1997). Recommended standards for reports dealing with lower extremity ischemia: revised version. J Vasc Surg.

[6] Latifi AN, Ibe U, Gnanaraj J (2019). A case report of atrial myxoma presenting with systemic embolization and myocardial infarction. Eur Heart J Case Rep.

[7] Ho KK, Barsoum R, Shepherd B, McGahan T (2020). Bilateral acute lower limb ischemia secondary to complete embolization of cardiac myxoma. J Vasc Surg.

[8] Aboyans V, Ricco JB, Bartelink ME (2018). 2017 ESC guidelines on the diagnosis and treatment of peripheral arterial diseases, in collaboration with the European Society for Vascular Surgery (ESVs). Rev Esp Cardiol (Engl Ed).

[9] Sadeghpour A, Alizadehasl A (2013). Showering emboli of an atrial mass. A fatal phenomenon. Res Cardiovasc Med.

[10] Vaidya Y, Sharma S (2026). Atrial myxoma. StatPearls [Internet].

[11] Vroomen M, Houthuizen P, Khamooshian A, Soliman Hamad MA, van Straten AH (2015). Long-term follow-up of 82 patients after surgical excision of atrial myxomas. Interact Cardiovasc Thorac Surg.

